# Deubiquitinase OTUD3: a double-edged sword in immunity and disease

**DOI:** 10.3389/fcell.2023.1237530

**Published:** 2023-09-26

**Authors:** Qiao Xu, Lan He, Shubing Zhang, Xiaotang Di, Hao Jiang

**Affiliations:** ^1^ Department of Cell Biology, School of Life Sciences, Central South University, Changsha, Hunan, China; ^2^ School of Biomedical Science, Hunan University, Changsha, Hunan, China; ^3^ Department of Biomedical Informatics, School of Life Sciences, Central South University, Changsha, Hunan, China

**Keywords:** deubiquitinase, OTUD3, cancer, immunity, disease

## Abstract

Deubiquitination is an important form of post-translational modification that regulates protein homeostasis. Ovarian tumor domain-containing proteins (OTUDs) subfamily member OTUD3 was identified as a deubiquitinating enzyme involved in the regulation of various physiological processes such as immunity and inflammation. Disturbances in these physiological processes trigger diseases in humans and animals, such as cancer, neurodegenerative diseases, diabetes, mastitis, etc. OTUD3 is aberrantly expressed in tumors and is a double-edged sword, exerting tumor-promoting or anti-tumor effects in different types of tumors affecting cancer cell proliferation, metastasis, and metabolism. OTUD3 is regulated at the transcriptional level by a number of MicroRNAs, such as miR-520h, miR-32, and miR101-3p. In addition, OTUD3 is regulated by a number of post-translational modifications, such as acetylation and ubiquitination. Therefore, understanding the regulatory mechanisms of OTUD3 expression can help provide insight into its function in human immunity and disease, offering the possibility of its use as a therapeutic target to diagnose or treat disease.

## 1 Introduction

Ubiquitination which is involved in the regulation of many eukaryotic signaling pathways is an important post-translational modification of proteins (Swatek and Komander, 2016). The process of ubiquitination modification is achieved by three enzymes, a ubiquitin-activating enzyme (E1), a ubiquitin-conjugating enzyme (E2), and ubiquitin ligase (E3) (Scheffner et al., 1995; Koegl et al., 1999; Rotin and Kumar, 2009). The ubiquitin molecule itself has eight ubiquitination modification sites, namely, seven lysine residues (K6, K11, K27, K29, K33, K48, and K63) and the N-terminal methionine residue (Met1) (Kulathu and Komander, 2012; Kwon and Ciechanover, 2017). The ubiquitination process can also be regulated by deubiquitinating enzymes (DUB), which cleave ubiquitin from substrate proteins and thus regulate the dynamic balance of ubiquitination and deubiquitination in the organism.

The human genome encodes about 100 DUBs, which are usually divided into two groups, the majority cysteine proteases, and metalloproteases. In the past, cysteine proteases were classified into four categories which are ubiquitin-specific protease (USP), ubiquitin C-terminal hydrolase (UCH), ovarian protease (OTU), and Machado-Joseph disease protease (MJD) (Nijman et al., 2005). In recent studies, two other categories MINDY (Abdul Rehman et al., 2016) and ZUFSP (Kwasna et al., 2018) were added to it. The JAB1/MPN/Mov34 metalloenzymes (JAMMs) with zinc-dependent properties are the only members of the metalloproteases family (Pan et al., 2022). Unlike USP family members that are non-specific for ubiquitin chain selection, OTU family members are linkage specific for different types of ubiquitin chains (Faesen Alex et al., 2011; Mevissen Tycho ET et al., 2013). Growing evidence that OTU subfamily members are associated with many human diseases such as cancer (Lin et al., 2019), immunity (Zhang et al., 2018), inflammation (Zhao et al., 2020), DNA damage repair (Kato et al., 2014), etc. The dysfunction that occurs with abnormal OTUD3 expression can also lead to the development of tumors, diabetes, and neurodegenerative diseases. In this paper, we first review the progress of research on the structure and molecular mechanisms and functions of OTUD3 in human and animal diseases and then describe the regulatory mechanisms of OTUD3 activity and expression.

## 2 OTUD3 is an OTUD-subfamily deubiquitinase

**FIGURE 1:**
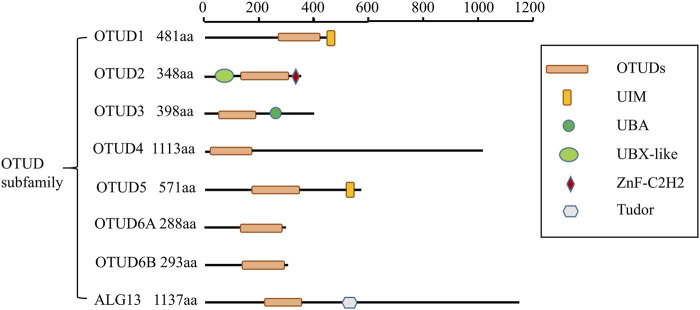
Schematic illustration of OTUD subfamily architectures.

The OTU structural domain was first identified in the *Drosophila* ovarian tumor gene from *Drosophila melanogaster* (Makarova et al., 2000). OTU-type DUBs can be divided into four subclasses based on the catalytic and other structural domains, the Otubains subfamily (OTUB1 and OTUB2), the OTUD subfamily (OTUD1, OTUD2, OTUD3, OTUD4, OTUD5, OTUD6A, OTUD6B, ALG13) (Figure 1), the A20-like OTUs subfamily (A20, OTUD7A, OTUD7B, Trabid, VCPIP1), and the OTULIN subfamily (Du et al., 2020).

In the OTU family, OTU domain-containing protein 3 (OTUD3) is described as a deubiquitinase that cleaves mainly Lys6 and Lys63 junctions and, to a lesser extent, Lys11 and Lys 48. OTUD3 is highly conserved evolutionarily and its protein structure consists of the catalytic OTU structural domain and the ubiquitin-associated (UBA) structural domain. The OTU structural domain contains a cysteine protease catalytic triad consisting of cysteine, aspartate, and histidine residues (Balakirev et al., 2003). The UBA structural domain is a small structural domain that consists of 40 residues with ubiquitin-binding functions (Buchberger, 2002). OTUD3 is localized on chromosome 1p36.13 and maintains its catalytic activity dependent on Cys76 (Du et al., 2019).

OTUD3 is expressed in all cells but at higher levels in neuron-derived tissues. And it is mainly distributed in the cytoplasmic interstitial space bound to microtubules in the cytoplasm and less in the nucleus. OTUD3 entry into the nucleus may depend on the interaction between itself and microtubule network dynamics. The substrate of OTUD3, p53, is present in the nucleus. P53 is associated with cellular microtubules and translocates to the nucleus in a kinesin-dependent manner (Marchenko et al., 2010). OTUD3 may enter the nucleus to mediate the process of deubiquitination of p53. Since a small fraction of OTUD3 is localized in the nucleus of non-stressed cells, it may also participate in the DNA repair pathway by counteracting Lys6 ubiquitination when it is not required. These are of course speculations and the function of OTUD3 into the nucleus needs to be further investigated (Job et al., 2022).

## 3 Biological functions of OTUD3

OTUD3 has many biological functions as a deubiquitinating enzyme that deubiquitinates a variety of substrates or interacts with related proteins (Table 1). Several recent studies have shown that OTUD3 is associated with many human and animal diseases, such as innate immunity, inflammation, tumors, diabetes, and clinical mastitis in cows.

**TABLE 1:** Summary of substrates for OTUD3.

Substrates	Ubiquitin chain types	Effects	References
Virus-related proteins			
MAVS	K63	Suppression of innate anti-RNA virus immunity	Zhang et al. (2020a)
RIG-I, MDA5	K63	Suppression of innate anti-RNA virus immunity	Cai et al. (2022)
cGAS	K48K27	Promotes DNA virus-triggered innate immunity	Lyu et al. (2023)
Ribosome-associated proteins			
eS10 and uS10	Not mentioned	Reduces RRub levels during stress recovery and limits RQC activation	Garshott et al. (2020)
Tumor suppressors			
PTEN	K6K11K27K48	Stabilizes PTEN and inhibits breast cancer progression	Yuan et al. (2015)
p53	Not mentioned	Deubiquitinates p53 from Mdm2-mediated ubiquitination and degradation, inhibiting breast cancer progression	Pu et al. (2020)
Tumor-promoting factors			
GRP78	Not mentioned	Stabilizes GRP78, which promotes lung cancer cell growth and migration	Du et al. (2019)
TRIM56	K48	Stabilizes TRIM56 and promotes lung adenocarcinoma	Lu (2021)
SOAT1	Not mentioned	Regulates SOAT1 protein stability and promotes the growth and metastasis of hepatocellular carcinoma cells	Gai et al. (2021)
ACTN4	Not mentioned	Stabilizes ACTN4 and promotes the growth and metastasis of hepatocellular carcinoma cells	Xie et al. (2021)
ZFP36	K48	Stabilizes ZFP36, thereby promoting the decay of VEGF-C mRNA and inhibiting lymphatic metastasis in esophageal cancer	Wang et al. (2021)
Metabolism-related proteins			
IRP2	K48	Stabilizes IRP2 protein, which is involved in iron metabolism	Jia et al. (2022)
Fortilin	Not mentioned	OTUD3 deubiquitinates fortilin and alleviated endoplasmic reticulum stress	Chen et al. (2023)
PPARδ	K48	Stabilizes various genes of PPARδ regulating glycolipid metabolism and oxidative phosphorylation (OXPHOS)	Zhou et al. (2022)

### 3.1 OTUD3 and immune response

**FIGURE 2:**
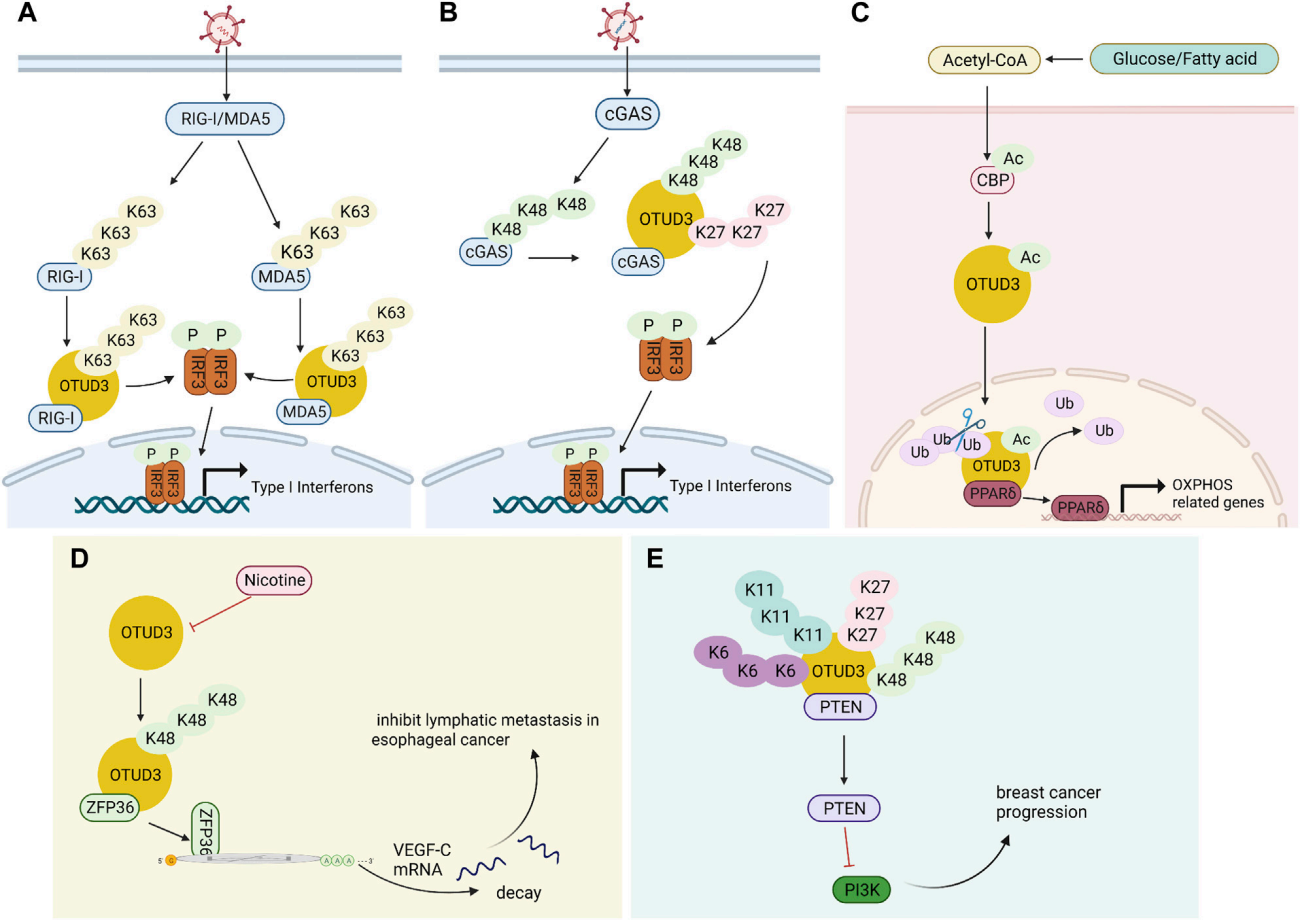
Multiple functions of OTUD3. **(A)** OTUD3 remove k63-linked ubiquitination of RIG-I and MDA5, thereby inhibiting the innate antiviral response triggered by RNA virus. **(B)** OTUD3 promotes DNA virus-triggered innate immunity. It binds cGAS and targets the Lys279 site for deubiquitination of k48- and k27-linked ubiquitination, thereby enhancing cGAS protein stability and DNA binding capacity. **(C)** The CBP promotes the acetylation of OTUD3 to its nuclear translocation, and OTUD3 regulates OXPHOS processes by stabilizing PPARδ. **(D)** OTUD3 stabilizes the k48-linked ubiquitinated ZFP36, and ZFP36 recruits RNA degradation complexes that mediate the decay of VEGF-C mRNA. **(E)** OTUD3-PTEN-PI3K axis may be the molecular mechanism of OTUD3 tumor suppression in breast cancer.

OTUD3 is a deubiquitinase dependent on its Lys129-acetylation, and acetylated OTUD3 hydrolyzes polyubiquitination of Lys63 on MAVS during resting state. During viral infection, SIRT1 deacetylates OTUD3, thereby rapidly inactivating OTUD3 and providing effective innate anti-RNA virus immunity (Zhang et al., 2020a). OTUD3 can also reduce the binding of RIG-I and MDA5 to viral RNA and MAVS by binding RIG-I and MDA5 and removing k63-linked ubiquitination, thereby inhibiting the innate antiviral response triggered by RNA virus (Figure 2A). Interestingly, in contrast to RNA virus, OTUD3 promotes DNA virus-triggered innate immunity. OTUD3 binds cGAS and targets the Lys279 site for deubiquitination of k48- and k27-linked ubiquitination, thereby enhancing cGAS protein stability and DNA binding capacity (Cai et al., 2022). OTUD3 can also promote the DNA binding ability and dimerization of cGAS, thereby enhancing the enzymatic activity of cGAS (Lyu et al., 2023) (Figure 2B).

OTUD3 not only participates in innate antiviral immunity, but also triggers inflammatory immunity. The expression of lipopolysaccharide-binding protein (LBP) and cyclooxygenase 2 (COX2) on the classical pathway of nigrostriatal NF-κB and the expression of C-C motif chemokine 19 (CCL19) and cell adhesion factor 1 (ICAM1) on the non-classical pathway were both upregulated after OTUD3 knockdown in mice. All of these suggest that OTUD3 may be involved in the neuroinflammatory response (Gao et al., 2021).

### 3.2 OTUD3 and ribosome-associated quality control (RQC) pathway

OTUD3 and USP21 are deubiquitinating enzymes that antagonize ZNF598-mediated 40S ubiquitination and limit RQC activation. Cells lacking USP21 or OTUD3 alter the activity of RQC, delaying the deubiquitination of eS10 causing the ribosome to retain its ubiquitin tag longer and more likely to stall and fail to continue reading mRNA (Garshott et al., 2020).

### 3.3 OTUD3 and cancers

#### 3.3.1 OTUD3 and cell proliferation

OTUD3 interacts with PTEN in the cytoplasm, deubiquitinates and stabilizes PTEN, ultimately inhibiting tumor growth and metastasis in breast cancer (Yuan et al., 2015). PTEN is a key negative regulator of the PI3K/p-AKT/mTOR pathway (Lee et al., 2018). Inhibition of PI3K/p-AKT/mTOR pathway activation thereby inhibits breast cancer progression with increased drug sensitivity (Song et al., 2012; Marcotte et al., 2016) (Figure 2E). The expression of OTUD3 is regulated by small non-coding RNAs. MiR-520h binds to the 3′-untranslated region (3′-UTR) of OTUD3 and inhibits OTUD3 protein expression, which in turn inhibits PTEN expression and ultimately activates the downstream pathway AKT. Activation of AKT pathway enhances proliferation, invasiveness and resistance to paclitaxel in breast cells (Geng et al., 2020). Similarly, MicroRNA 32 (miR-32) can directly target the 3′-untranslated region (3′-UTR) of OTUD3 to inhibit OTUD3 expression, which can promote proliferation and motility of colon cancer cells and inhibits apoptosis (Jin et al., 2019). OTUD3 inhibits BC cell proliferation and makes BC cells more sensitive to chemotherapeutic drug-induced apoptosis by directly interacting with p53 through the amino-terminal OTU region to deubiquitinate it from murine double minute 2 (Mdm2)-mediated ubiquitination and degradation (Pu et al., 2020).

#### 3.3.2 OTUD3 and cell metastasis

Carboxyl terminus of Hsc70-interacting protein CHIP is the ubiquitin ligase of OTUD3. OTUD3 is stabilized in lung cancer cell lines by the deletion of CHIP, and the level of intracellular GRP78 increases, thus promoting lung cancer cell invasion and tumor metastasis (Zhang et al., 2020b). A small molecule OTUD3 inhibitor named OTUDin3 has been reported for the treatment of non-small cell lung cancer. In NSCLC, OTUDin3 exhibited significant anti-proliferative and pro-apoptotic effects by inhibiting the deubiquitination activity of OTUD3 (Zhang et al., 2023). OTUD3 mediates Alpha-actinin 4 ACTN4 deubiquitination in HCC to stabilize it and promote hepatocellular carcinoma cell growth and metastasis (Xie et al., 2021). In addition, ACTN4 promotes metastasis to lSymph nodes in colorectal cancer as well as induces epithelial mesenchymal transition and tumorigenesis in cervical cancer, and it can be inferred that OTUD3 is also involved in these processes by regulating ACTN4 (Honda et al., 2005; An et al., 2016). OTUD3 also deubiquitinates k48-linked ubiquitination to stabilize ZFP36 ring finger protein (ZFP36), which ZFP36 recruits RNA degradation complexes to mediate the decay of VEGF-C mRNA. Downregulation of OTUD3 expression by Nicotine in esophageal cancer leads to downregulation of ZFP36, which induces VEGF-C production and promotes lymphatic metastasis in esophageal cancer (Wang et al., 2021) (Figure 2D). TRIM56 acts as a ubiquitin ligase to increase the ubiquitination level of lung adenocarcinoma cancer-promoting factor OTUD3 and promote its ubiquitin-proteasome pathway degradation. OTUD3 also feedback regulates TRIM56 and is able to remove the Lys48-linked polyubiquitin chain on TRIM56 and stabilize its protein expression. TRIM56 acts as a tumor suppressor, inhibiting the migration and invasion of lung adenocarcinoma (Lu, 2021).

#### 3.3.3 OTUD3 and cell metabolism

OTUD3 is highly expressed in human lung cancer tissues and is associated with poor patient survival. OTUD3 interacts with the glucose-regulated protein GRP78 to deubiquitinate and stabilize GRP78, thereby inhibiting lung cancer cell growth and migration (Du et al., 2019). OTUD3 increases SOAT1 protein stability by specifically removing the polyubiquitination modification of sterol O-acyltransferase 1 (SOAT1), dependent on its deubiquitinating enzyme activity (Gai et al., 2021). SOAT1 affects the growth and migration of hepatocellular carcinoma cells by promoting the synthesis of cholesterol and consequently (Jiang et al., 2019).

#### 3.3.4 Mysterious role of OTUD3 in cancers

OTUD3 has been elucidated to exert pro- or oncogenic functions in tumors by deubiquitinating and stabilizing the expression of related proteins. However, the expression and mechanism of action of OTUD3 in other tumors remain unknown. PTEN and OTUD3 protein expression was significantly lower in C6 cells compared to primary astrocytes, and survival time was longer in patients with high OTUD3-expressing gliomas than in patients with low-expressing gliomas. It was concluded only that the low expression of OTUD3 in glioma cells may be involved in gliomagenesis, but the mechanism behind this is unclear (Liu et al., 2020). Similarly, upon genomic analysis of human cancers, OTUD3 locus deletion may be responsible for the development of multiple squamous cell carcinomas (SCCs). However, the molecular basis of OTUD3 in SCC as a tumor suppressor has not been fully elucidated. It is speculated that OTUD3 function may be impaired in SCCs, destabilizing the tumor suppressor PTEN and thus activating the AKT pathway to cause cancer (Gatti et al., 2020).

OTUD3 appears to have both pro- and anti-cancer effects in cancer. Whether it exerts a cancer-promoting or cancer-suppressing effect may depend on its substrate protein. If the substrate protein is a tumor suppressor, OTUD3 stabilizes the expression of the substrate protein by deubiquitination and is also a tumor suppressor. However, more clinical evidence and mechanistic studies are still needed to further elucidate the role of OTUD3 in cancer in order to target it for antitumor therapy.

### 3.4 OTUD3 in other diseases

#### 3.4.1 Neurological diseases

In recent years there have been many studies on the factors involved in the degeneration of dopaminergic neurons in PD, and iron deposition has received much attention (Moreau et al., 2018). Iron is essential for cellular biosynthesis, metabolic activity, and the balance of iron uptake, efflux and storage in cells is regulated by iron regulatory proteins regulating IRPs (Hentze et al., 2010). Intracellular IRP2 is degraded via the ubiquitin-proteasome pathway. OTUD3 acts as a deubiquitinating enzyme for IRP2, promoting the removal of k48-linked polyubiquitination of IRP2 in the cytoplasm and stabilizing IRP2 protein in an iron-independent manner. Thus OTUD3 deficiency leads to disruption of iron metabolism (Jia et al., 2022). OTUD3 deficiency also leads to dopaminergic neuronal damage, which may be another reason for the appearance of Parkinson’s symptoms. OTUD3 regulates the level of fortilin ubiquitination, affects endoplasmic reticulum stress, and inhibits dopaminergic neuronal apoptosis (Chen et al., 2023).

#### 3.4.2 Diabetes

The OTUD3 c.863G>A (rs78466831) mutation was identified in the obesity and diabetes family, a mutation that reduces the stability and catalytic activity of OTUD3. Otud3^−/−^ mice show a significant impairment of energy metabolism and are susceptible to obesity and metabolic disorders. To gain more insight into the function of OTUD3, RNA-seq analysis was performed on the mutant and wild type. GSEA analysis (Subramanian et al., 2005) showed that OTUD3 loss-of-function-associated genes scored high in diabetes, PPAR signaling pathway, glycolipid metabolism, and fatty acid degradation. Glucose and fatty acid stimulation leads to increased levels of acetyl coenzyme a and increased levels of acetylation of CREB-binding protein (CBP) (Wellen et al., 2009; Cordero-Herrera et al., 2017). The acetyltransferase CBP promotes OTUD3 acetylation to its nuclear translocation, where OTUD3 regulates glycolipid metabolism and oxidative phosphorylation (OXPHOS) processes through the stabilization of the metabolism-associated factor peroxisome-proliferator-activated receptor delta (PPARδ) (Zhou et al., 2022) (Figure 2C). In addition, a comparison of the prevalence of rs78466831 in patients with type 2 diabetes mellitus T2DM in different regions revealed that the rs78466831 mutation was strongly associated with T2DM in a province in eastern China and had a higher risk of developing diabetes retinopathy DR (Liu et al., 2022). It is possible that mutations in specific amino acids in OTUD3 lead to changes in protein conformation (Kar et al., 2017), which in turn leads to a much higher risk of developing diabetes.

#### 3.4.3 Tissue inflammation

Defective OTUD3 gene triggers tissue inflammation in humans and animals. OTUD3 is a susceptibility gene for Crohn’s disease and ulcerative colitis (Jung et al., 2023). The three RNF186-OTUD3-PLA2G2E genes are located on chromosome 1p36, and 1p36 contains two different ulcerative colitis risk SNPs (rs1317209 and rs6426833). Previous studies have shown that OTUD3 transcripts are higher in immune tissues and lymphocytes than in CD14-positive cells, suggesting that OTUD3 regulates the immune response (McGovern et al., 2010), and that mutations in it may be responsible for developing ulcerative colitis. Further studies found that the approximately 8 kb SNP rs4654903, located upstream of OTUD3, is closer to the OTUD3 position and has a stronger signal than the two previous SNPs, similar to Caucasian and Asian (Yang et al., 2013). A 200 bp deletion in the Copy number variant regions (CNVRs) of OTUD3 (BTA2) in Holstein-Friesian cows was positively correlated with the incidence of clinical mastitis (CM) (Szyda et al., 2019).

#### 3.4.4 Recurrent spontaneous abortion

Long-stranded noncoding RNA SNHG6 and OTUD3 are upregulated and miR-101-3p is downregulated in recurrent spontaneous abortion (RSA) patients. In RSA, SNHG6 binds to and negatively regulates miR101-3p expression and OTUD3 is a miR101-3p target. SNHG6 reduces trophoblast cell viability and migration capacity by regulating miR101-3p/OTUD3 and increases trophoblast apoptosis. SNHG6/miR-101-3p/OTUD3 could be a potential target for the prevention or treatment of RSA (Zhao et al., 2022).

OTUD3 is involved in the regulation of iron metabolism and glucose metabolism through deubiquitination of metabolism-related proteins. When its deficiency occurs, it induces metabolism-related diseases. Also, OTUD3 is a susceptibility gene for inflammatory diseases. Therefore, uncovering more information about OTUD3 can help to understand the pathogenesis of these diseases and to treat them.

## 4 Regulation of OTUD3 activity and expression

As with most deubiquitinating enzymes, the activity and expression of OTUD3 is regulated by mechanisms of action at the transcriptional and post-translational levels (Table 2).

**TABLE 2:** Summary of the upstream regulator for OTUD3.

Upstream regulator	Regulation method	Mechanism	References
Proteins			
SIRT1	Deacetylated OTUD3	Inactivates OTUD3 to provide effective innate immunity against RNA virus	Zhang et al. (2020a)
CBP	Acetylated OTUD3	Acetylates OTUD3, allowing it to enter the nucleus	Zhou et al. (2022)
CHIP	Ubiquitinated OTUD3	Degrades OTUD3, inhibits OTUD3-GRP78 signaling axis and suppresses tumor metastasis in lung cancer	Zhang et al. (2020b)
TRIM56	Ubiquitinated OTUD3	Degrades OTUD3, inhibits invasion and migration of lung adenocarcinoma	Lu (2021)
RNAs			
miR-520h	Binding 3′-UTR of OTUD3	Inhibit the expression of OTUD3 in breast cancer cells, thereby enhancing the resistance of breast cancer cells to paclitaxel	Geng et al. (2020)
miR-32	Binding 3′-UTR of OTUD3	Inhibition of OTUD3 expression promotes cell proliferation, motility and inhibits apoptosis in colon cancer cells	Jin et al. (2019)
miR101-3p	Binding 3′-UTR of OTUD3	Inhibition of OTUD3 expression increases trophoblast cell viability and migration ability and reduces trophoblast cell apoptosis	Zhao et al. (2022)
miR-34c-5p	Not mentioned	Negatively targeted OTUD3 which enhanced proliferation, angiogenesis, and migration in HPMECs	He et al. (2023)

### 4.1 Transcription regulation

**FIGURE 3:**
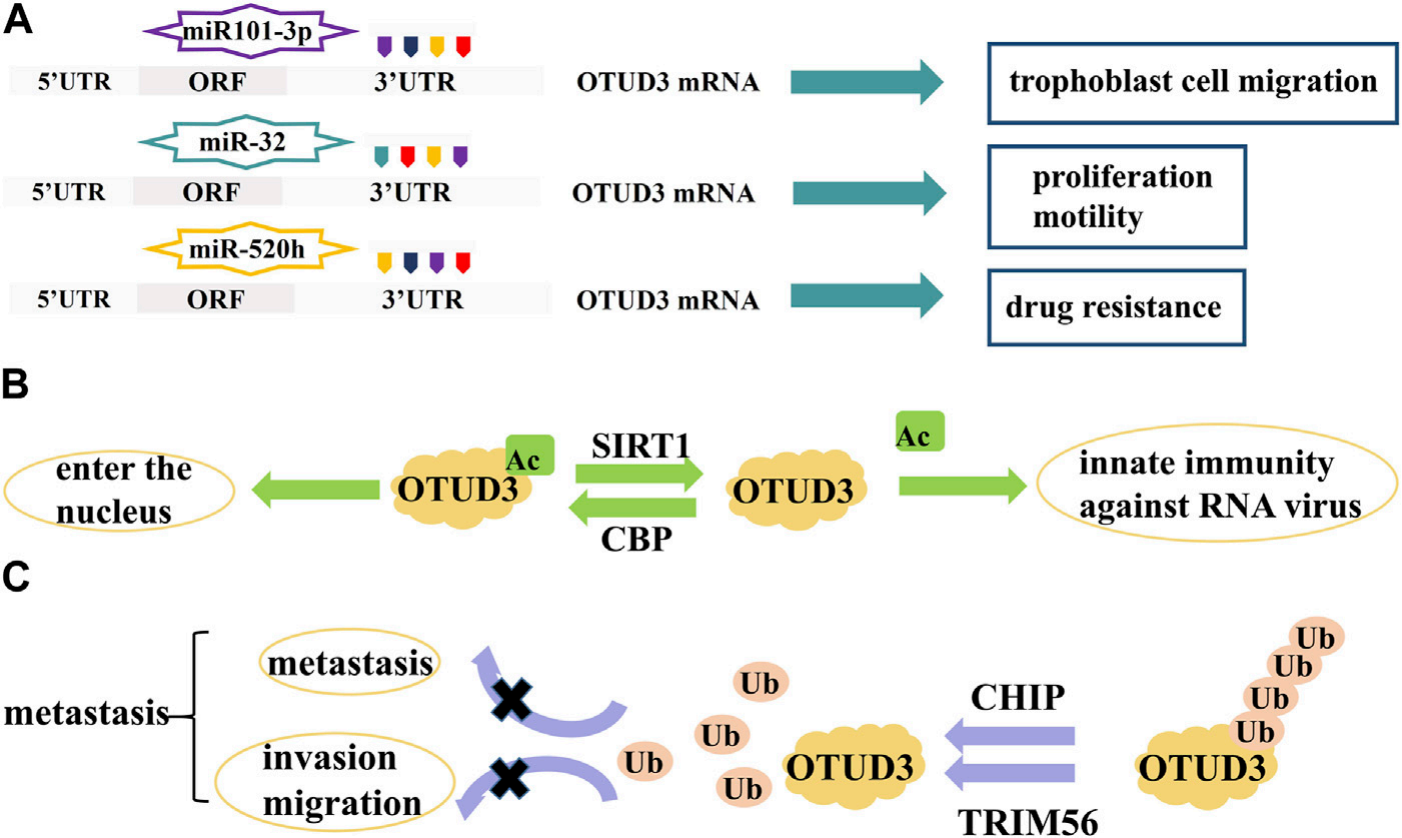
Proteins and RNAs regulating OTUD3 activity and expression. **(A)** MicroRNAs regulate the transcription of OTUD3. **(B)** SIRTI and CBP regulate the acetylation of OTUD3. **(C)** CHIP and TRIM56 regulate the ubiquitination of OTUD3.

OTUD3 is regulated at the transcriptional level by a number of microRNAs which target the 3′-UTR region of OTUD3 mRNA and are involved in the regulation of cell proliferation and apoptosis. For example, miR-520h binds to OTUD3 to inhibit the expression of OTUD3 protein in breast cancer cells, which in turn enhances the resistance of breast cancer cells to paclitaxel (Geng et al., 2020). MiR-32 also inhibits the expression of OTUD3 and promotes cell proliferation and motility and inhibits apoptosis in colon cancer cells (Jin et al., 2019). In addition, miR101-3p restored the viability and migration ability of trophoblast cells by inhibiting OTUD3 expression, while reducing trophoblast cell apoptosis (Zhao et al., 2022). In Bronchopulmonary dysplasia (BPD) mouse, miR-34c-5p negatively targeted OTUD3 which enhanced proliferation, angiogenesis, and migration in human pulmonary microvascular endothelial cells (HPMECs) (He et al., 2023) (Figure 3A).

### 4.2 Posttranslational modifications

OTUD3 is regulated by a number of post-translational modifications, such as acetylation and ubiquitination.

#### 4.2.1 Acetylation

OTUD3 activity is dependent on its Lys129-acetylation. During the onset of innate anti-RNA virus immunity, OTUD3 is regulated by SIRT1 deacetylation and activity is lost, resulting in effective resistance to RNA viruses (Zhang et al., 2020a). OTUD3 acetylation can also affect its cellular localization. Normally, OTUD3 is localized in the cytoplasm and can enter the nucleus after acetylation. For example, the acetyltransferase CBP promotes OTUD3 acetylation for its nuclear translocation, allowing OTUD3 to deubiquitinate and stabilize the intranuclear metabolism-associated transcription factor PPARδ thereby affecting gene expression related to glycolipid metabolism and oxidative phosphorylation (OXPHOS) (Zhou et al., 2022) (Figure 3B).

#### 4.2.2 Ubiquitination

The ubiquitin ligase CHIP ubiquitinates OTUD3 to degrade it, and inhibition of the OTUD3-GRP78 signaling axis reduces tumor metastasis in lung cancer (Zhang et al., 2020b). OTUD3 can also be ubiquitinated by TRIM56, thereby inhibiting invasion and migration of lung adenocarcinoma (Marcotte et al., 2016) (Figure 3C).

## 5 Discussion and conclusion

This paper reviews the physiopathological roles of OTUD3 in human diseases such as innate immunity, inflammatory response, tumorigenesis, diabetes, and neurodegenerative diseases, and explores in depth the regulatory mechanisms of its activity and expression. Interestingly, OTUD3 is a double-edged sword for tumors, playing a pro- or oncogenic role in different types of tumors, yet the regulatory mechanisms of OTUD3 expression, localization and function in these tumors are still not fully explored. Understanding these mechanisms can help develop drugs targeting OTUD3 to inhibit cancer progression and provide new ideas for cancer therapy.

OTUD3 mRNA levels were significantly lower in breast cancer tissues compared to paraneoplastic tissues, while prognostic risk assessment models for OTUD3 showed patients with strong OTUD3 mRNA expression had a better prognosis (Qi et al., 2019). This could serve as evidence that OTUD3 acts as a tumor suppressor in breast cancer. During tumorigenesis, the PI3K/p-AKT/mTOR pathway is regulated by OTUD3, and PTEN is a key regulatory protein of the PI3K/p-AKT/mTOR pathway. Since OTUD3 is a specific cytoplasmic deubiquitinase of PTEN, the OTUD3-PTEN-PI3K axis may also be the molecular mechanism of OTUD3 tumor suppression in breast cancer, glioma and SCC. In addition to exerting cancer-inhibiting effects, OTUD3 also exerts cancer-promoting functions in liver and lung cancers, promoting the growth of liver and lung cancer cells. OTUD3 is like a double-sided person, which side it exhibits may depend on its partner substrate proteins. If the substrate protein is a tumor suppressor, OTUD3 stabilizes the expression of the substrate protein by deubiquitination and also functions as a cancer suppressor. In antiviral immune responses, OTUD3 is similarly two-sided in that it inhibits the innate antiviral immune response to RNA viruses but promotes the innate antiviral immune response to DNA viruses. Most of the proteins identified to date are consistent in regulating innate antiviral immunity in response to DNA and RNA viruses, either promoting or inhibiting, with few opposing effects found (Zhang et al., 2020c). Deubiquitinases that remove K63 or K48-linked polyubiquitin chains exerting opposite effects, like OTUD3, have rarely been reported. The opposite role of OTUD3 in the innate antiviral immune response may be consistent with its opposite mechanism of action in cancer, both depending on the function of its substrate.

OTUD3 activity is influenced by post-translational modifications and its activity is dependent on Lys129-acetylation. As a deubiquitinating enzyme, OTUD3 itself can be degraded by ubiquitination mediated by two E3 ubiquitin ligases, CHIP and TRIM56. In addition to acetylation and ubiquitination modifications, other post-translational modifications such as phosphorylation, hydroxylation and hypusination (Kar et al., 2021; Park et al., 2022; Ziegler et al., 2022) were not reported.

We still have not found the commonality of OTUD3 substrate proteins and the real reason for OTUD3 entry into the nucleus. Therefore, it is necessary to further explore the molecular mechanisms of OTUD3 in diseases and its unique functions. In the future, structural modeling analysis of OTUD3 protein can be performed using proteomics technology and bioinformatics to predict its substrate proteins and discover their structural commonalities, which can help to elucidate the regulatory mechanisms of OTUD3 in human diseases. As kinases and phosphatases are main drug targets for various cancers (Nemec et al., 2019), researchers attempted to find kinases that are up- or downregulated in the OTUD3 pathway, and unfortunately did not find any. However, a small molecule inhibitor, OTUDin3, which directly targets OTUD3, has been found. Given the oncogenic role of OTUD3 in many cancers, if more small molecule inhibitors of OTUD3 for cancer therapy are fortunately screened and identified to inhibit its expression or activity, it is expected to be a promising therapeutic strategy for the treatment of a range of human tumors.
